# Sensitization to oil palm pollen associates with risks and severity of allergic diseases

**DOI:** 10.1016/j.waojou.2023.100853

**Published:** 2024-01-09

**Authors:** Yang Yie Sio, Gallego Allaine Victoria Nanong, Jie Ann Lim, Sri Anusha Matta, Yee-How Say, Keng Foo Teh, Yi Ru Wong, Smyrna Moti Rawanan Shah, Kavita Reginald, Fook Tim Chew

**Affiliations:** a Department of Biological Sciences, National University of Singapore, Singapore; b Department of Biomedical Science, Faculty of Science, Universiti Tunku Abdul Rahman (UTAR) Kampar Campus, Kampar, Perak, Malaysia; c Department of Biological Sciences, School of Medical and Life Sciences, Sunway University, Malaysia

**Keywords:** Allergy, Immunoglobulin E, Pollen, Asthma, Rhinitis

## Abstract

**Background:**

*Elaeis guineensis* (Ela g, oil palm) pollen is one of the most predominant species of inhalant allergens in the tropical Southeast Asia region; however, its association with the manifestation of allergic diseases remains largely unexplored. This study aimed to determine the sensitization pattern of oil palm pollen and associate this with the risk and severity of allergic diseases.

**Methods:**

Participants were recruited as a part of the Singapore and Malaysia cross-sectional genetic and epidemiological study (SMCSGES). Two independent cohorts were recruited: *n* = 564 serum samples were collected and serological assessment was performed against a panel of 16 crude inhalant allergens including house dust mite, pet, insect, pollen, and fungal allergens; *n* = 13 652 Singapore/Malaysia Chinese young adults were recruited and skin prick test was used to assess oil palm sensitization, which was tested for its association with the risk and severity of asthma, allergic rhinitis (AR), and atopic dermatitis (AD).

**Results:**

The sensitization rate of oil palm pollen is 9.6% in the *n* = 564 Singapore/Malaysia cohort. In the *n* = 13 652 Singapore/Malaysia Chinese cohort, oil palm sensitization significantly associates with increased risks of asthma (*p* = 1.34x10^−4^), AR (*p* = 2.91x10^−13^), and AD (*p* = 6.95x10^−7^). Asthmatic patients with oil palm sensitization have increased risks of wheezing (*p* = 0.00995), nocturnal cough (*p* = 0.0122), and exacerbations (*p* = 0.00139) in the past 12 months. AR patients with oil palm sensitization also have an increased risk of developing moderate-to-severe symptoms (*p* = 0.00113).

**Conclusions:**

We have identified significant associations of oil palm sensitization with increased risks, exacerbations, and the severity of symptoms of allergic diseases in the tropical Southeast Asian region (Singapore/Malaysia).

## Introduction

Allergic diseases including asthma, allergic rhinitis (AR), and atopic dermatitis (AD) are common disorders with increased global prevalence over recent decades.^1^, ^2^, ^3^ Sensitizations to airborne inhalant allergens such as house dust mite (HDM), pollens, and fungal spores are implicated as major causes of these allergic diseases.^4^, ^5^, ^6^ In the tropical environment of Southeast Asia, an abundance of indoor HDM allergens was found in the home environment year-round.^7^ Studies also revealed a spectrum of pollen and fungal airspora that was uniquely present in the tropical environment with high allergenicity.^6^^,^^8^^,^^9^ Of these allergens, the pollen of *Elaeis guineensis* (Ela g, oil palm) was one of the most predominant types of outdoor allergens in tropical countries,^8^^,^^10^ especially in Singapore during the Northeast Monsoon season (December to March).^8^ This is likely due to the presence of about 6.4 and 12.7 million hectares of oil palm plantations in Malaysia and Indonesia respectively.^11^ The oil palm pollen can be disseminated from these countries to Singapore by wind dispersal, due to their close proximities.^12^

Oil palm sensitization is an important risk factor of asthma and AR in the Southeast Asian tropical region.^6^^,^^13^ Multiple oil palm pollen proteins have been characterized and showed IgE reactivity, including a 31-kDa palm pollen glycoprotein (Ela g Bd 31 K),^14^ a pectin esterase-like oil palm pollen protein designated as ELGU1,^15^ as well as a polygalacturonase-like protein.^16^ While the allergenicity of these proteins has been assessed previously, the specific role of oil palm sensitization in the manifestation of allergic diseases remains to be investigated.

Among the airborne inhalant allergens commonly present in the tropical environment of Southeast Asia, HDM sensitization was frequently associated with the risk and severity of allergic airway diseases.^5^^,^^17^^,^^18^ Fungal sensitization was also correlated with the susceptibility and severity of asthma and AR in the Singapore/Malaysia population.^4^^,^^6^ However, the association between oil palm sensitization and the risk and severity of allergic diseases in this region is not well characterized. Here, we report associations of oil palm sensitization with the risk, exacerbation, and severity of allergic diseases including asthma, AR, and AD in the Singapore and Malaysia populations. Serum specific IgE (SSIgE) titers against a panel of 16 common inhalant allergens were assessed in a cohort of n = 564 Singapore/Malaysia young adults. In a separate cross-sectional cohort of *n* = 13 652 Singapore/Malaysia young adults of Chinese ethnicity, oil palm sensitization was assessed using skin prick test and associated with the risks, exacerbations, and severity of symptoms of allergic diseases. This allows understanding of the role of oil palm sensitization in the manifestation of allergic diseases in the tropical environment of Singapore and Malaysia.

## Materials and methods

### Study design

This present study belongs to a part of an ongoing Singapore and Malaysia cross-sectional genetic and epidemiological study (SMCSGES). Participants were recruited from this ongoing epidemiological collection in Singapore and Malaysia universities: National University of Singapore, Singapore (NUS, from Aug 2005 to Sep 2019), University Tunku Abdul Rahman, Malaysia (UTAR, from Feb 2016 to Oct 2018), and Sunway University, Malaysia (SU, Nov 2019). The recruitment of participants from NUS has been previously described.^5^^,^^19^, ^20^, ^21^, ^22^

Two independent cross-sectional cohorts were reported in this study: 1) serum samples of *n* = 564 participants were collected from UTAR and SU for serological assessment using the immune-dot blot approach; 2) detailed information on demographics, medical history, and severity of allergic diseases of *n* = 13 652 Chinese individuals were collected from NUS, UTAR, and SU using an investigator-administered questionnaire. Participants from both cohorts underwent a skin prick test (SPT), using a panel of 4 types of allergens, including 2 HDM species (*Dermatophagoides pteronyssinus* and *Blomia tropicalis*), oil palm pollen (*Elaeis guineensis*), and a fungus species (*Curvularia lunata*). A positive SPT was defined as having a wheal of at least 3 mm in diameter 15 min after the skin prick. The SPT was performed during the recruitment process. These allergens used in the SPT were the most common inhalant allergen present in the Singapore environment with high allergenicity, as demonstrated previously.^6^^,^^23^ Sensitizations to HDM and fungal allergens have already been discussed in our previous publications.^4^^,^^5^ In this manuscript, we focus on sensitization against oil palm pollen due to its high abundance in the atmosphere of the tropical Southeast Asia region.^23^ The demographics of both cohorts are summarized in Table S1.

### Cloning, expression, and purification of Ela g profilin

The Ela g profilin gene was cloned into a modified pET28b (+) vector and overexpressed as a 6 × His-tag fusion protein. The construct was transformed into *Escherichia coli* strain BL21 (DE3) to produce the Ela g profilin protein. Transformed bacteria cells were grown overnight in Luria Bertani (LB, Bio Basic Inc., Canada) containing kanamycin (100 μg/mL) at 37°C. Bacteria cultures were then inoculated and grown in 1L LB until OD600 reached 0.6. Recombinant protein expression was induced by the addition of 0.5 mM isopropyl β-d-1-thiogalactopyranoside (IPTG, 1st Base, Singapore) to the bacterial cultures, which were grown at 37°C for 4 h before harvesting by centrifugation at 5465*g*. The pellet was resuspended in binding buffer (20 mM Tris–HCl pH 7.9, 0.5 M NaCl, 20 mM imidazole, and 6 M urea) and lysed by sonication at an amplitude of 38% for 15 min (30 s pulse on and 30 s pulse off) followed by centrifugation at 14 000 g. The supernatant containing the profilin protein was filter-sterilized using a 0.22 μm PVDF membrane filter and then applied onto an Ni–NTA-coupled HisTrap HP 5 ml column (GE Healthcare, UK) which had been pre-equilibrated with 20 mL binding buffer. The Ela g profilin protein was eluted using a linear gradient of washing buffer (20 mM Tris–HCl pH 7.9, 0.5 M NaCl, 0.5 M imidazole, and 6 M urea). Fraction containing soluble Ela g profilin protein was used for subsequent immuno-dot blot assay.

### Immuno-dot blot assay

SSIgE titers were measured using the immuno-dot blot approach. To collect serum samples, 10 mL of whole blood were obtained from the participants and then centrifuged at 2600 g and 4°C for 15 min. Subsequently, SSIgE titers against 16 common inhalant allergens were measured, including 3 HDM species (*Dermatophagoides pteronyssinus, Dermatophagoides farinae*, and *Blomia tropicalis*), dog (native *Canis familiaris*, Can f 1 protein), cat (native *Felis domesticus*, Fel d 1 protein), American cockroach (*Periplaneta americana*), and German cockroach (*Blatella germanica*), 4 fungal species (*Aspergillus* sp., *Cladosporium* sp., *Curvularia* spp., *Penicillium* sp.), oil palm (*Elaeis guineensis*), chloridoids (*Cynodon dactylon*), panicoids (*Sorghum halepense*), pooids (*Phleum pratense*, *Festuca pratensis*, and *Lolium perenne*), and weeds (*Brassica* spp., *Ambrosia artemisifolia*, and *Helianthus annus*) were measured using the immuno-dot blot assay, as described previously.^24^, ^25^, ^26^, ^27^ SSIgE titers against these allergens were also previously measured by our group using the ImmunoCAP system approved by the United States Food and Drug Administration (FDA),^5^ and these prior findings were in concordance with the present immuno-dot blot results. We also performed the immune-dot blot assay to assess SSIgE titers against the Ela g profilin recombinant protein. All immuno-dot blot measurements were conducted at the same time to avoid batch variation. A positive sensitization to the tested allergen was defined as SSIgE titers >0.35IU/ml.

### Survey questionnaires and disease definition

In the *n* = 13 652 Singapore/Malaysia Chinese cohort, participants were requested to complete an investigator-administered questionnaire collecting information on demographics and medical history, which was based on the Allergic Rhinitis Impact on Asthma (ARIA)^28^ guidelines and International Study of Asthma and Allergies in Childhood (ISAAC)^29^ questionnaire. Ethnicity was self-reported through survey questionnaires and confirmed in a previously performed principal component analysis.^30^

Asthma was defined as ever having asthma positively diagnosed by a physician. AR was defined as having at least 2 major AR-related symptoms that include nasal congestion, rhinorrhea, nasal itching, and sneezing (based on 2008 guidelines set by the ARIA consortium).^28^ Moderate-severe AR was defined as having at least 1 of the following AR-related symptoms: disturbed sleep, impaired daily activities including sport, leisure, impaired work and school, and troublesome symptoms (based on 2008 guidelines set by the ARIA consortium).^28^ AD was defined as having a persistent itchy rash that affected flexural areas.

### Statistical analysis

Association analyses between oil palm sensitization and all allergic disease-related phenotypes were performed using logistic regression analysis in the R program version 3.6.1 (R Foundation for Statistical Computing, Vienna, Austria), and *p* < 0.05 was considered as statistically significant. Spearman correlation analyses of SSIgE and total IgE levels were also performed using the R program.

## Results

### Allergen sensitization profile in the Singapore/Malaysia population

**Fig. 1:**
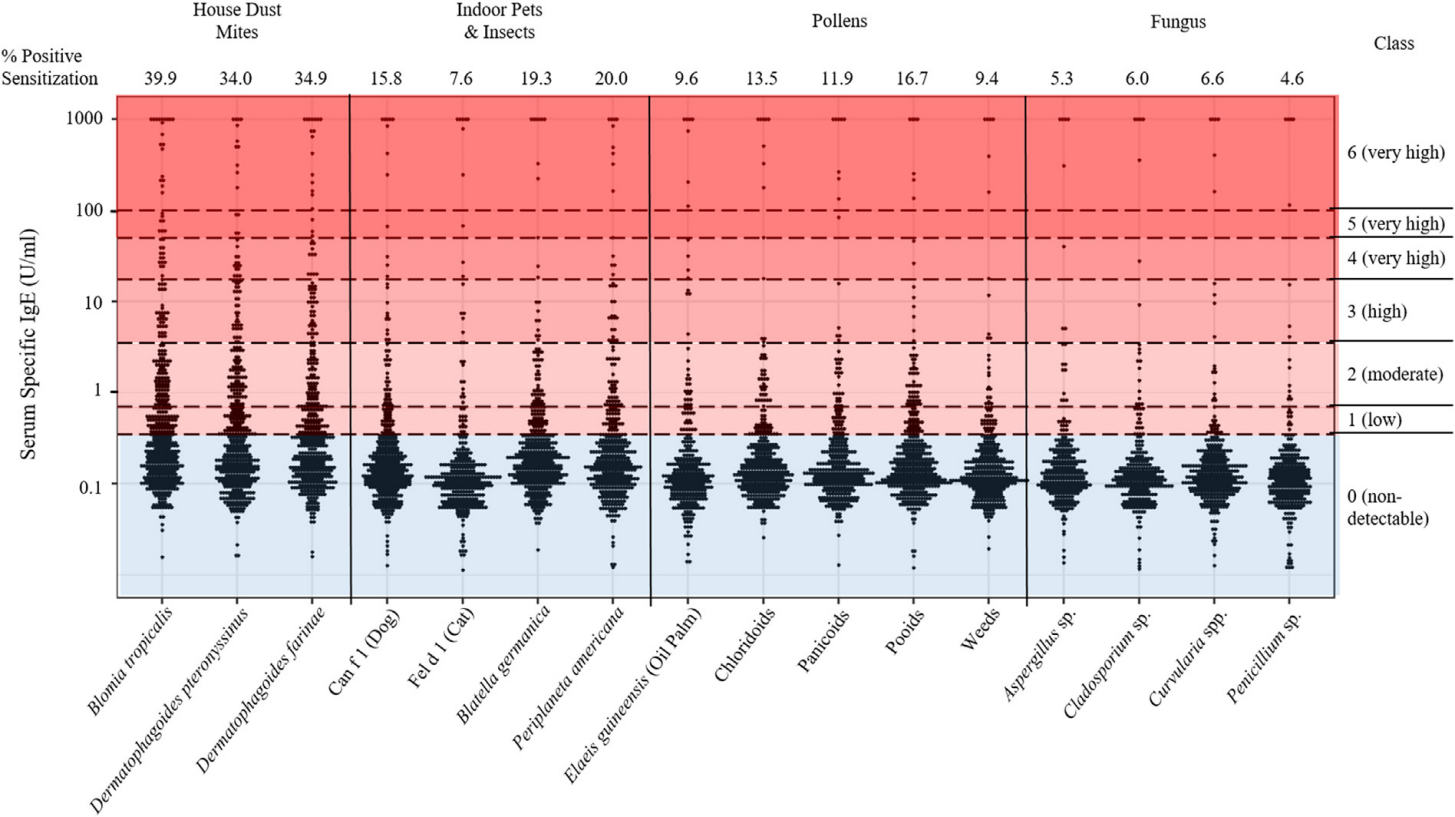
**Allergen sensitization profile of 16 common inhalant allergens in the Singapore/Malaysia population**. Serum specific immunoglobulin-E (SSIgE) titers (as IU/ml) against each allergen are indicated, using a cohort of 564 Singapore/Malaysia young adults. Assessed allergens include 3 HDM species (*Dermatophagoides pteronyssinus*, *Dermatophagoides farinae*, and *Blomia tropicalis*), dog (native *Canis familiaris*, Can f 1 protein), cat (native *Felis domesticus*, Fel d 1 protein), American cockroach (*Periplaneta americana*), and German cockroach (*Blatella germanica*), 4 fungal species (*Aspergillus* sp., *Cladosporium* sp., *Curvularia* spp., *Penicillium* sp.), oil palm (*Elaeis guineensis*), chloridoids (*Cynodon dactylon*), panicoids (*Sorghum halepense*), pooids (*Phleum pratense*, *Festuca pratensis*, *Lolium perenne*), and weeds (*Brassica* spp., *Ambrosia artemisifolia*, *Helianthus annus*). SSIgE titers were assessed using the immuno-dot blot approach.

To assess the allergen sensitization profile in the Singapore/Malaysia population, we collected *n* = 564 serum samples from the SMCSGES cohort (age: 21.16 ± 3.49, 49.1% male, table S1). SSIgE titers against 16 common allergens were measured using the immunoblot approach and positive sensitization was defined as SSIgE titers >0.35IU/ml (Fig. 1). Sensitization to HDM allergens was the most prevalent in this cohort (*Blomia tropicalis*: 39.9%, *Dermatophagoides pteronyssinus*: 34.0%, and *Dermatophagoides farinae*: 34.9%), with 49.3% of individuals sensitized to the allergen of at least 1 HDM species tested (Fig. 1). Also, SSIgE titers against these 3 HDM allergens were highly correlated (Spearman's Rho ranged from 0.797 to 0.857, all *p* < 0.0001; Fig. S1). For allergens of indoor pets and insects, the frequencies of sensitization to the dog (Can f 1), cat (Fel d 1), German cockroach (*Blatella germanica*), and American cockroach (*Periplaneta americana*) were 15.8%, 7.6%, 19.3%, and 20.0% respectively (Fig. 1).

In this cohort, the sensitization rates of 5 pollen allergens were measured, including oil palm (*Elaeis guineensis*, 9.6%), chloridoids (*Cynodon dactylon*, 13.5%), panicoids (*Sorghum halepense*, 11.9%), pooids (*Phleum pratense*, *Festuca pratensis*, and *Lolium perenne*, 16.7%), and weeds (*Brassica* spp., *Ambrosia artemisifolia*, and *Helianthus annus*, 9.4%, Fig. 1). SSIgE titers against all of these pollen allergens were also correlated with each other (Spearman's Rho ranged from 0.505 to 0.800, all *p* < 0.0001; Fig. S2). By contrast, SSIgE titers against 4 common types of fungal allergens were generally lower than all the other types of allergen tested (*Aspergillus* sp.: 5.3%, *Cladosporium* sp.: 6.0%, *Curvularia* sp.: 6.6%, and *Penicilium* sp.: 4.6%, Fig. 1). Pairwise correlations were observed across serum IgE titers specific to all fungal allergens tested (Spearman's Rho ranged from 0.391 to 0.765, all *p* < 0.0001; Fig. S3). This is in agreement with previous findings.^4^

### Correlation between total IgE and serum sIgE levels specific to HDM and oil palm pollen

**Fig. 2:**
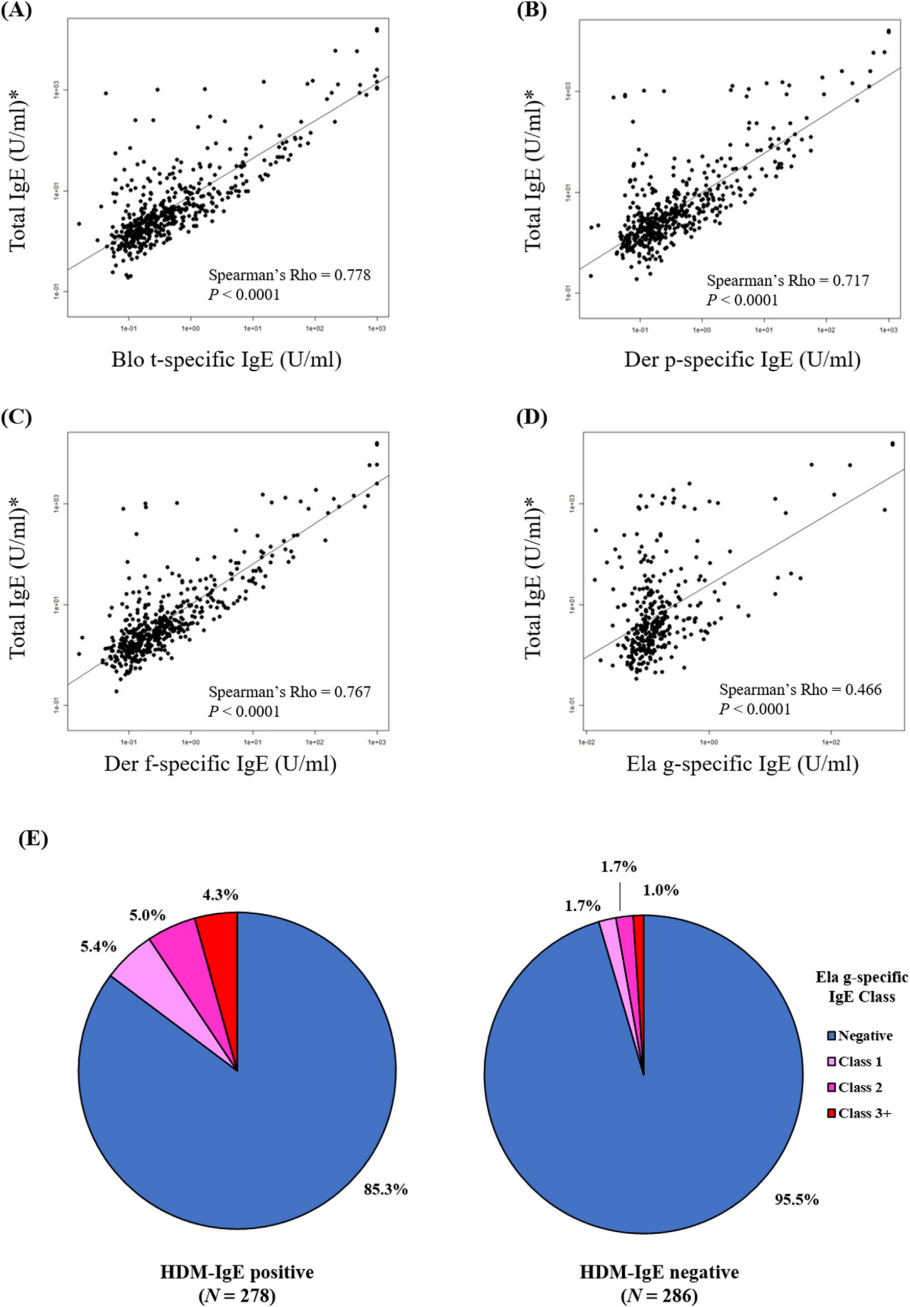
**Correlation analysis of allergen sensitization and serum total IgE in the Singapore/Malaysia population**. Correlation between serum total IgE titers and serum specific IgE (SSIgE) titers against (A) *Blomia tropicalis* (Blo t), (B) *Dermatophagoides pteronyssinus* (Der p) (C) *Dermatophagoides farinae* (Der f), and (D) *Elaeis guineensis* (Ela g, oil palm pollen) were analyzed using Spearman correlation test. Serum total IgE titers were estimated by the summation of SSIgE titers against 16 types of allergen, including 2 HDM species (Blo t, Der p, Der f), dog (native *Canis familiaris*, Can f 1 protein), cat (native Fel d 1 protein), American cockroach (*Periplaneta americana*), and German cockroach (*Blatella germanica*), 4 fungal species (*Aspergillus* sp., *Cladosporium* sp., *Curvularia* spp., *Penicillium* sp.), oil palm (*Elaeis guineensis*), chloridoids (*Cynodon dactylon*), panicoids (*Sorghum halepense*), pooids (*Phleum pratense*, *Festuca pratensis*, *Lolium perenne*), and weeds (*Brassica* spp., *Ambrosia artemisifolia*, *Helianthus annus*). (E) Percentage of individuals with positive oil palm sensitization among either HDM-sensitized or non-HDM-sensitized individuals. A positive sensitization to the tested allergen was defined as sIgE titers >0.35IU/ml. The immuno-dot blot approach was used to measure serum sIgE titers using a cohort of *n* = 564 Singapore/Malaysia young adults.

HDM sensitization was previously correlated to the atopy condition, as well as the serum total IgE titer in the tropical Southeast Asian population.^5^ To confirm these findings, SSIgE titers against all 16 common allergens in this present cohort were combined and used as an estimation of the total IgE titer. As shown in Fig. 2, SSIgE titers against 3 HDM species were all strongly correlated with the total IgE titer (Spearman's Rho ranged from 0.717 to 0.778, all p < 0.0001). A significant correlation was also observed between the SSIgE titer against oil palm and the total IgE titer (Spearman's Rho = 0.466, p < 0.0001). Thus, both HDM and oil palm sensitizations might be indicative of a higher total IgE titer, which also suggest a correlation with atopy in the Singapore/Malaysia population. Besides, individuals sensitized to HDM also showed a higher sensitization rate to oil palm pollen (14.7%), as compared to the HDM-sIgE-negative individuals (4.4% sensitized to oil palm pollen). This suggests polysensitization against both HDM and oil palm allergens was more prevalent in the Singapore/Malaysia population, as compared to monosensitization against either type of these allergens.

### Association between oil palm sensitization and risks of allergic diseases in the Singapore/Malaysia population

To evaluate the association between oil palm sensitization and the risks of allergic diseases in the Singapore/Malaysia population, we recruited *n* = 13 652 Singapore/Malaysia young adults of Chinese ethnicity from the SMCSGES cohort (age: 21.96 ± 5.004, 41.5% male, table S1). The prevalence of asthma, AR, and AD were 17.6%, 39.9%, and 17.9%, respectively. In this cohort, sensitization rates of 4 common inhalant allergens were measured using SPT, including *Blomia tropicalis* (43.2%), *Dermatophagoides pteronyssinus* (40.3%), *Elaeis guineensis* (oil palm pollen, 4%), and *Curvularia lunata* (1.4%). Oil palm sensitization was associated with increased risks of asthma (*p* = 1.34 x 10^−4^, OR = 1.47, 95% CI: 1.20–1.78), AR (*p* = 2.91 x 10^−13^, OR = 2.01, 95% CI: 1.67–2.43), and AD (*p* = 6.95 x 10^−7^, OR = 1.65, 95% CI: 1.35–2.00, Table 1). IgE sensitization to oil palm pollen was also associated with having at least one allergic disease (asthma, AR, and/or AD, *p* = 7.86 x 10^−11^, OR = 2.08, 95% CI: 1.68–2.61, Table 1). Oil palm sensitization was associated with a higher risk of disease multimorbidity (affected by more than one allergic disease, *p* = 8.35 x 10^−16^, OR = 2.80, 95% CI: 2.19–3.61, Table 1).

**Table 1:** Association between *Elaeis guineensis* (oil palm) pollen sensitization and the risk of allergic diseases in the Singapore/Malaysia Chinese population.

	Total (*n* = 13 652)			
	*Elaeis guineensis* Positive SPT	*Elaeis guineensis* Negative SPT	*P*-value	OR (95% CI)
	Case/Control (%Case)	Case/Control (%Case)		
**Asthma**^a^	152/417 (26.7)	1713/7235 (19.1)	**1.34 x 10**^**−**^**^4^∗**	1.47 (1.20–1.78)
**Allergic Rhinitis (AR)**^b^	331/188 (63.8)	3901/4379 (47.1)	**2.91 x 10**^**−**^**^13^∗**	2.01 (1.67–2.43)
**Atopic Dermatitis (AD)**^c^	150/386 (28)	1757/7104 (19.8)	**6.95 x 10**^**−**^**^7^∗**	1.65 (1.35–2.00)
**Single allergic disease only (Asthma-only or AR-only or AD-only)**	240/110 (68.6)	3678/2841 (56.4)	**3.77 x 10**^**−**^**^6^∗**	1.75 (1.38–2.22)
**At least one allergic disease (Asthma or AR or AD)**	421/110 (79.3)	5385/2841 (65.5)	**7.86 x 10**^**−**^**^11^∗**	2.08 (1.68–2.61)
**More than one allergic disease (disease multimorbidity)**	181/110 (62.2)	1707/2841 (37.5)	**8.35 x 10**^**−**^**^16^∗**	2.80 (2.19–3.61)
**Asthma** + **AR**	96/148 (39.3)	893/3553 (20.1)	**9.67 x 10**^**−**^**^11^∗**	2.51 (1.89–3.31)
**Asthma** + **AD**	45/270 (14.3)	424/5289 (7.4)	**2.23 x 10**^**−**^**^5^∗**	2.07 (1.46–2.87)
**AR** + **AD**	102/137 (42.7)	948/3448 (21.6)	**4.49 x 10**^**−**^**^14^∗**	2.83 (2.16–3.70)
**Asthma** + **AR** + **AD**	31/110 (22)	279/2841 (8.9)	**2.34 x 10**^**−**^**^6^∗**	2.84 (1.81–4.32)

CI: confidence interval; OR: odds ratio; SPT: skin prick test. All data were evaluated based on skin prick test results of the *Elaeis guineensis* (oil palm) pollen allergen in a cross-sectional cohort of Singapore/Malaysia Chinese individuals (*n* = 13 652). *P*-value, odds ratio, and 95% CI were calculated using a logistic regression analysis with adjustment for age and gender.

∗Logistic *p* < 0.05 is considered as significant.

a Allergic asthma case was defined as ever having asthma positively diagnosed by a physician (Based on ISAAC questionnaire). Non-asthma control was defined as no symptoms and history of asthma.

b Allergic rhinitis (AR) case is defined by 2 or more self-reported rhinitis symptoms. Non-AR control was defined as no symptoms and history of AR.

c Atopic Dermatitis (AD) case is defined as having a persistent itchy rash that affected flexural areas. Non-AD control was classified based on no symptoms and history of AD

Further, most of these sensitization-to-disease associations remained significant after we performed stratified analyses by using only the HDM-sensitized individuals (Table 2). In this sub-cohort of HDM sensitized individuals (*n* = 8089, Table S1), oil palm sensitization was significantly associated with increased risks of AR (*p* = 0.00142, OR = 1.40, 95% CI: 1.14–1.72) and AD (*p* = 1.18 x 10^−4^, OR = 1.52, 95% CI: 1.22–1.87); however, its association with an increased risk of asthma was marginally significant (*p* = 0.0528, OR = 1.22, 95% CI: 1.00–1.50, Table 2). Also, oil palm sensitization was associated with increased risks of developing at least one allergic disease (asthma, AR, and/or AD, *p* = 0.00728, OR = 1.40, 95% CI: 1.10–1.79) and a higher risk of disease multimorbidity (*p* = 2.07 x 10-5, OR = 1.81, 95% CI: 1.38–2.38, Table 2). These findings suggest the associations of oil palm sensitization with allergy are independent of HDM sensitization.

**Table 2:** Association between *Elaeis guineensis* (oil palm) pollen sensitization and the susceptibility of allergic diseases among house dust mite-sensitized individuals in the Singapore/Malaysia Chinese population.

	HDM-sensitized individuals (*n* = 8089)			
	*Elaeis guineensis* Positive SPT	*Elaeis guineensis* Negative SPT	*P*-value	OR (95% CI)
	Case/Control (%Case)	Case/Control (%Case)		
**Asthma**^a^	146/350 (29.4)	1443/4312 (25.1)	0.0528	1.22 (1.00–1.50)
**Allergic Rhinitis (AR)**^b^	296/155 (65.6)	3075/2145 (58.9)	**0.00142∗**	1.4 (1.14–1.72)
**Atopic Dermatitis (AD)**^c^	134/329 (28.9)	1235/4409 (21.9)	**1.18 x 10**^**−**^**^4^∗**	1.52 (1.22–1.87)
**Single allergic disease only (Asthma-only or AR-only or AD-only)**	206/90 (69.6)	2651/1303 (67)	0.212	1.18 (0.91–1.54)
**At least one allergic disease (Asthma or AR or AD)**	376/90 (80.7)	4076/1303 (75.8)	**0.00728∗**	1.40 (1.10–1.79)
**More than one allergic disease (disease multimorbidity)**	170/90 (65.4)	1425/1303 (52.2)	**2.07 x 10**^**−**^**^5^∗**	1.81 (1.38–2.38)
**Asthma + AR**	94/121 (43.7)	796/1647 (32.6)	**5.91 x 10**^**−**^**^4^∗**	1.67 (1.24–2.23)
**Asthma + AD**	43/224 (16.1)	361/3064 (10.5)	**0.00365∗**	1.68 (1.17–2.36)
**AR + AD**	93/113 (45.1)	772/1677 (31.5)	**2.84 x 10**^**−**^**^5^∗**	1.87 (1.39–2.51)
**Asthma + AR + AD**	30/90 (25)	252/1303 (16.2)	**0.00944∗**	1.81 (1.14–2.80)

CI: confidence interval; HDM: house dust mite; OR: odds ratio; SPT: skin prick test. All data were evaluated based on skin prick test results of the *Elaeis guineensis* (oil palm) pollen allergen in a cross-sectional cohort of Singapore/Malaysia Chinese individuals with positive sensitization to house dust mite allergens (*n* = 8089). *P*-value, odds ratio, and 95% CI were calculated using a logistic regression analysis with adjustment for age and gender.

∗Logistic *p* < 0.05 is considered as significant.

a Allergic asthma case was defined as ever having asthma positively diagnosed by a physician (Based on ISAAC questionnaire). Non-asthma control was defined as no symptoms and history of asthma.

b Allergic rhinitis (AR) case is defined by 2 or more self-reported rhinitis symptoms. Non-AR control was defined as no symptoms and history of AR.

c Atopic Dermatitis (AD) case is defined as having a persistent itchy rash that affected flexural areas. Non-AD control was classified based on no symptoms and history of AD

### Association between Elaeis guineensis sensitization and severity of allergic diseases in the Singapore/Malaysia population

In the *n* = 13 652 Singapore/Malaysia Chinese cohort (Table S1), we next evaluated if oil palm sensitization associates with the severity of allergic diseases. Among the asthmatic patients (*n* = 2405), oil palm sensitization significantly associates with the presence of recent (past 12 months) asthma-related symptoms and exacerbations, including wheezing (*p* = 0.00995, OR = 1.68, 95% CI: 1.12–2.46), nocturnal cough (*p* = 0.0122, OR = 1.62, 95% CI: 1.10–2.35), daytime asthma attack (*p* = 5.03 x 10^−4^, OR = 2.07, 95% CI: 1.36–3.10), nighttime asthma attack (*p* = 1.15 x 10^−6^, OR = 2.77, 95% CI: 1.82–4.13), general practitioners/specialist visit for asthma (*p* = 0.00112, OR = 2.11, 95% CI: 1.33–3.28), and asthma-related hospitalization (*p* = 0.0189, OR = 3.55, 95% CI: 1.11–9.64, Table 3). Collectively, we also observed significant association between oil palm sensitization and any asthma exacerbation event in the past 12 months (*p* = 0.00139, OR = 2.07, 95% CI: 1.31–3.21, Table 3). Among the AR-affected individuals (*n* = 5453), oil palm sensitization was associated with the presence of itchy nose symptoms in past 12 months (*p* = 0.0181, OR = 1.41, 95% CI: 1.07–1.90, Table 3). Further, AR patients sensitized against oil palm pollen were more likely to be affected by moderate-to-severe AR symptoms as compared to non-sensitized patients (*p* = 0.00113, OR = 1.53, 95% CI: 1.19–1.98, Table 3).

**Table 3:** Associations of *Elaeis guineensis* (oil palm) pollen sensitization with the severity and frequency of symptoms of allergic diseases in the Singapore/Malaysia Chinese population.

	Total (*n* = 13 652)			
	*Elaeis guineensis* Positive SPT	*Elaeis guineensis* Negative SPT	*P*-value	OR (95% CI)
	Case/Control (%Case)	Case/Control (%Case)		
**Asthma-related symptoms**^a^				
Wheezing	39/113 (25.7)	291/1422 (17)	**0.00995**∗	1.68 (1.12–2.46)
Wheezing with exercise	26/126 (17.1)	210/1503 (12.3)	0.0806	1.49 (0.93–2.30)
Nocturnal cough	42/110 (27.6)	332/1381 (19.4)	**0.0122**∗	1.62 (1.10–2.35)
**Asthma-related exacerbations**^a^				
Daytime Asthma Attack	35/117 (23)	214/1499 (12.5)	**5.03 x 10**^**−**^**^4^**∗	2.07 (1.36–3.1)
Nighttime Asthma Attack	37/115 (24.3)	180/1533 (10.5)	**1.15 x 10**^**−**^**^6^**∗	2.77 (1.82–4.13)
School Absence	7/145 (4.6)	80/1633 (4.7)	0.614	0.8 (0.31–1.81)
GP/Specialist Visits	32/120 (21.1)	194/1519 (11.3)	**0.00112**∗	2.11 (1.33–3.28)
A&E Admission	7/145 (4.6)	48/1665 (2.8)	0.400	1.46 (0.56–3.36)
Hospitalization	5/147 (3.3)	15/1698 (0.9)	**0.0189**∗	3.55 (1.11–9.64)
Any Exacerbation Event^b^	33/119 (21.7)	206/1507 (12)	**0.00139**∗	2.07 (1.31–3.21)
**AR-related symptoms**^c^				
Itchy nose	266/62 (81.1)	2950/929 (76.1)	**0.0181**∗	1.41 (1.07–1.90)
Sneezing	300/29 (91.2)	3531/362 (90.7)	0.642	1.10 (0.75–1.67)
Runny nose	279/52 (84.3)	3268/615 (84.2)	0.896	0.98 (0.72–1.35)
Nose blockage	265/65 (80.3)	3119/757 (80.5)	0.579	0.92 (0.70–1.24)
**Severity of AR symptoms**^d^				
Mild, *n* (%)	86 (26)	1367 (35)		
Moderate-to-severe, *n* (%)	245 (74)	2534 (65)	**0.00113**∗	1.53 (1.19–1.98)

A&E: accident and emergency department of a hospital; AR: allergic rhinitis; CI: confidence interval; GP: general practitioner; OR: odds ratio; SPT: skin prick test. All data were evaluated based on skin prick test results of the *Elaeis guineensis* (oil palm) pollen allergen in a cross-sectional cohort of Singapore/Malaysia Chinese individuals (*n* = 13 652). *P*-value, odds ratio, and 95% CI were calculated using a logistic regression analysis with adjustment for age and gender.

∗*p* < 0.05 is considered as significant.

a Asthma-related symptoms and exacerbation: cases are defined as asthma-affected individuals with the presence of asthma-related symptoms or exacerbations in the past 12 months. Controls are defined as asthma-affected individual with the absence of respective symptoms or exacerbations in the past 12 months.

b Any exacerbation event includes school absence, GP/specialist visits, A&E admission, or hospitalization due to asthma exacerbation.

c AR-related symptoms: cases are defined as AR-affected individuals with the presence of AR-related symptoms or exacerbations in the past 12 months. Controls are defined as AR-affected individual with the absence of respective symptoms in the past 12 months.

d Severity of AR symptoms: Moderate-to-severe AR was defined as AR-affected individuals having at least one of the following disturbances due to AR-related symptoms in the past 12 months: disturbed sleep, impaired daily activities including sport, leisure, impaired work and school, and troublesome symptoms. Mild AR was defined as AR-affected individuals with the absence of these disturbances

Association between oil palm sensitization and the severity of asthma and AR was also significant in the stratified cohort of HDM-sensitized individuals (*n* = 8089, Table 4). In the sub-cohort of HDM sensitized asthma patients (*n* = 1842), oil palm sensitization was associated with the presence of recent (past 12 months) wheezing (*p* = 0.0116, OR = 1.67, 95% CI: 1.11–2.46), nocturnal cough (*p* = 0.0150, OR = 1.62, 95% CI: 1.09–2.36), daytime asthma attack (*p* = 0.00107, OR = 2.02, 95% CI: 1.31–3.04), nighttime asthma attack (*p* = 5.38 x 10-6, OR = 2.64, 95% CI: 1.72–3.98), general practitioners/specialist visit for asthma (*p* = 0.00207, OR = 2.08, 95% CI: 1.29–3.27), asthma-related hospitalization (*p* = 0.0305, OR = 3.22, 95% CI: 1.01–8.77), and any asthma exacerbation event (*p* = 0.00256, OR = 2.03, 95% CI: 1.27–3.19, Table 4). In the sub-cohort of HDM-sensitized AR patients (*n* = 3849), oil palm sensitization was associated with the presence of recent (past 12 months) itchy nose symptoms (*p* = 0.0415, OR = 1.38, 95% CI: 1.02–1.89) and moderate-to-severe AR symptoms (*p* = 0.00266, OR = 1.53, 95% CI: 1.16–2.02, Table 4). Overall, these results suggest an independent influence of oil palm pollen sensitization on the symptoms and severity of asthma and AR.

**Table 4:** Associations of *Elaeis guineensis* (oil palm) pollen sensitization with the severity and frequency of symptoms of allergic diseases among house dust mite-sensitized individuals in the Singapore/Malaysia Chinese population.

	HDM-sensitized individuals (*n* = 8089)			
	*Elaeis guineensis* Positive SPT	*Elaeis guineensis* Negative SPT	*P*-value	OR (95% CI)
	Case/Control (%Case)	Case/Control (%Case)		
**Asthma-related symptoms**^a^				
Wheezing	39/107 (26.7)	261/1182 (18.1)	**0.0116**∗	1.67 (1.11–2.46)
Wheezing with exercise	26/120 (17.8)	180/1263 (12.5)	0.0581	1.55 (0.97–2.41)
Nocturnal cough	41/105 (28.1)	286/1157 (19.8)	**0.0150**∗	1.62 (1.09–2.36)
**Asthma-related exacerbations**^a^				
Daytime Asthma Attack	34/112 (23.3)	189/1254 (13.1)	**0.00107**∗	2.02 (1.31–3.04)
Nighttime Asthma Attack	36/110 (24.7)	162/1281 (11.2)	**5.38 x 10**^**−**^**^6^**∗	2.64 (1.72–3.98)
School Absence	6/140 (4.1)	73/1370 (5.1)	0.389	0.66 (0.24–1.57)
GP/Specialist Visits	31/115 (21.2)	172/1271 (11.9)	**0.00207**∗	2.08 (1.29–3.27)
A&E Admission	7/139 (4.8)	45/1398 (3.1)	0.428	1.43 (0.54–3.31)
Hospitalization	5/141 (3.4)	15/1428 (1)	**0.0305**∗	3.22 (1.01–8.77)
Any Exacerbation Event^b^	32/114 (21.9)	183/1260 (12.7)	**0.00256**∗	2.03 (1.27–3.19)
**AR-related symptoms**^c^				
Itchy nose	239/54 (81.6)	2361/702 (77.1)	**0.0415**∗	1.38 (1.02–1.89)
Sneezing	268/27 (90.8)	2801/269 (91.2)	0.933	0.98 (0.66–1.52)
Runny nose	249/47 (84.1)	2634/426 (86.1)	0.293	0.84 (0.61–1.18)
Nose blockage	240/55 (81.4)	2493/563 (81.6)	0.647	0.93 (0.69–1.28)
**Severity of AR symptoms**^d^				
Mild, *n* (%)	73 (24.7)	1022 (33.2)		
Moderate-to-severe, *n* (%)	223 (75.3)	2053 (66.8)	**0.00266**∗	1.53 (1.16–2.02)

A&E: accident and emergency department of a hospital; AR: allergic rhinitis; CI: confidence interval; GP: general practitioner; HDM: house dust mite; OR: odds ratio; SPT: skin prick test. All data were evaluated based on skin prick test results of the *Elaeis guineensis* (oil palm) pollen allergen in a cross-sectional cohort of Singapore/Malaysia Chinese individuals with positive sensitization to house dust mite allergens (*n* = 8089). *P*-value, odds ratio, and 95% CI were calculated using a logistic regression analysis with adjustment for age and gender.

∗*p* < 0.05 is considered as significant.

a Asthma-related symptoms and exacerbation: cases are defined as asthma-affected individuals with the presence of asthma-related symptoms or exacerbations in the past 12 months. Controls are defined as asthma-affected individual with the absence of respective symptoms or exacerbations in the past 12 months.

b Any exacerbation event includes school absence, GP/specialist visits, A&E admission, or hospitalization due to asthma exacerbation.

c AR-related symptoms: cases are defined as AR-affected individuals with the presence of AR-related symptoms or exacerbations in the past 12 months. Controls are defined as AR-affected individual with the absence of respective symptoms in the past 12 months.

d Severity of AR symptoms: Moderate-to-severe AR was defined as AR-affected individuals having at least one of the following disturbances due to AR-related symptoms in the past 12 months: disturbed sleep, impaired daily activities including sport, leisure, impaired work and school, and troublesome symptoms. Mild AR was defined as AR-affected individuals with the absence of these disturbances

Lastly, we also examined the associations between oil palm sensitization and severity of AD symptoms among AD-affected individuals (*n* = 2447); however, the results were not significant (Table S2).

### Sensitization to the Ela g profilin allergen is not associated with oil palm pollen sensitization in the Singapore/Malaysia population

Accumulating evidences have demonstrated profilin as one of the most important tree pollen panallergens (reviewed in Asam et al^31^). Therefore, we sought to investigate the prevalence of Ela g profilin sensitization in the present study population. Serological assessment of SSIgE titers against Ela g profilin was performed on *n* = 228 recruited subjects from the serological assessment cohort (Table S1). As shown in Table S3, the frequency of SSIgE sensitization against Ela g profilin (>0.35IU/ml) was not significantly different between oil palm pollen-sensitized subjects (17.4%) and oil palm pollen non-sensitized subjects (7.3%, *p* = 0.076, Table S3 and Fig. S4). This suggests the Ela g profilin is not a major allergen of the oil palm pollen. Further, we also examined the associations between Ela g profilin sensitization and the risk of allergic diseases in this cohort, however, the results were not significant (data not shown).

## Discussion

Sensitizations to HDM and fungal allergens were previously associated with the risk and severity of allergic diseases in the tropical Southeast Asian population.^4^, ^5^, ^6^^,^^13^^,^^17^^,^^18^ However, the association of pollen sensitization with the risk and severity of allergic diseases in this region was not well characterized. To address this, the present study first detected a substantial sensitization rate (9.4–16.7%) of 5 types of tree and grass pollen allergens (*Elaeis guineensis*, chloridoids, panicoids, pooids, and weeds) in the Singapore/Malaysia population, as well as a high cross-reactivity among these pollen allergens. In a separate cross-sectional cohort of *n* = 13 652 Singapore/Malaysia young adults of Chinese ethnicity, oil palm sensitization was significantly associated with the risks, exacerbations, and severity of symptoms of allergic diseases.

The tropical climatic condition of Singapore and Malaysia favors the growth of vegetation all year round, resulting in a rich and diverse spectrum of fungi, fern spore, and pollen presented in this environment.^8^^,^^23^ In Singapore, a high abundance of oil palm pollen was detected in the atmosphere, especially during the Northeast Monsoon season (December to March).^8^ Due to the abundance of this pollen in the atmosphere, a high frequency of oil palm sensitization (11.8%–48%) was reported in the Singapore population.^6^ This has also been evaluated in the present study, using a large-scale cross-sectional cohort of Singapore/Malaysia young adults that is independent of the previous cohort. We report an oil palm sensitization rate of 9.6% in Singapore and Malaysia, similar to the rate reported in Thailand (8.3% among pediatric asthmatics),^17^ but lower than the rate reported in Indonesia (22.43%).^13^

Studies have suggested oil palm sensitization may associate with the development of allergy-related phenotypes in the Asian population. For instance, a higher frequency of oil palm sensitization was found in both Singaporean and Indonesian patients affected by asthma and/or AR.^6^^,^^13^ Also, the incidence of asthma and AR was significantly higher among palm tree garden workers, compared to office workers in Iran.^32^ Our current results have thus confirmed these findings, where increased risks, exacerbation, and severity of symptoms of asthma, AR, and AD were observed in oil palm sensitized individuals. Significantly increased nasal symptoms were also observed in persistent AR patients 5 min after nasal challenge with *Elaeis guineensis* (unpublished data), using a previously reported approach performed by our group.^33^

In our work, skin prick test was performed using a panel of 4 types of allergens: 2 house dust mite (HDM) species (*Dermatophagoides pteronyssinus* and *Blomia tropicalis*), oil palm pollen (*Elaeis guineensis*), and a fungus species (*Curvularia lunata*). These allergens were previously shown to be the most common indoor and outdoor inhalant allergen presented in the Singapore environment with high allergenicity.^5^^,^^6^ We have previously shown that allergic response is largely dominated by HDM allergens: HDM sensitization was strongly associated with the risk and severity of asthma, AR, and AD.^5^ At least 70% of Singaporean/Malaysian individuals were skin prick test (SPT) positive against HDM,^5^ whereas about 4% of individuals were SPT positive against oil palm pollen (Table S1). Despite this, the present stratified analysis has also shown oil palm pollen sensitization can influence the manifestation of allergic diseases, independent of HDM sensitization. This suggested both indoor (HDM) and outdoor (oil palm pollen) allergens contribute independently in the development of allergic disorders in this region. Further, in this Singapore/Malaysia population, polysensitization against HDM and oil palm pollen is more prevalent and is associated with an increased risk and severity of allergic diseases compared to monosensitization (unpublished data). This will be discussed in a separate publication.

Accumulating evidences have demonstrated profilin as one of the most important tree pollen panallergens (reviewed in Asam et al^31^). A study in Spain has showed 56% of date palm pollen-allergic patients had a specific IgE-reactivity to natural profilin Pho d 2.^34^ Positive SPT to the date palm pollen profilin was also observed among 25% of pollen-allergic children in Italy.^35^ In the present study, the frequency of SSIgE sensitization against Ela g profilin (>0.35IU/ml) in the oil palm-sensitized individuals was not significantly higher than the non-sensitized controls (*p* = 0.076). This suggests the Ela g profilin is not a major allergen of the oil palm pollen. Individuals sensitized to oil palm pollen may show sensitizations towards other sources of novel tree pollen allergens.

A seasonal effect on the atmospheric concentration of oil palm pollen was previously observed.^8^ Its concentration peaks during the Northeast Monsoon season (December to March).^8^ The present study has not investigated whether this can influence the association between oil palm sensitization and allergy-related phenotype, due to the limitation of a cross-sectional study. Further study using a longitudinal cohort with multiple follow-up time points across different seasons within a year might help to address this question. Besides, we cannot determine whether age can influence the allergenicity of oil palm in our study population because the recruited cross-sectional cohort comprised mostly young adults (age: 21.96 ± 5.004 in the large-scale epidemiological assessment cohort, Table S1). Given the pollen sensitization rate was previously shown to increase from childhood to adulthood,^6^ future studies involving participants across age groups are therefore required to validate and investigate this. As a limitation of this study, we were unable to directly measure the total IgE levels in serum due to the restrictions in the volume of serum available. Total IgE levels were derived from the summation of SSIgE levels of individual allergens. This might confound the correlation analysis between total IgE and SSIgE levels. However, in this population, we have previously demonstrated that measured total IgE levels were correlated with the ssIgE levels of house dust mite (*Blomia tropicalis* and *Dermatophagoides pteronyssinus*).^5^ These correlations have now been replicated in this present study (Fig. 2A–C). A direct measurement of total IgE should also be performed in the future study to validate our present findings.

Overall, the present findings emphasized.an important role of oil palm pollen allergen in the manifestation of allergic disorders in this region. In allergy patients with oil palm pollen sensitization, implementation of allergen-specific immunotherapy might help to reduce morbidity. This approach has been proven to be effective in the treatment of moderate-severe seasonal AR.^36^ Allergen avoidance has also been suggested to be effective in managing asthma-related phenotypes.^12^^,^^37^ By reducing the exposure of oil palm pollen allergen during the Northeast Monsoon season (December to March), the prevalence of oil palm allergy can be reduced in this population.

## Conclusion

In conclusion, the present study has provided an up-to-date sensitization profile of common inhalant allergen and the associations of oil palm sensitization with the risk and severity of allergic diseases in the tropical environment of Singapore and Malaysia. The associations of oil palm sensitization with allergic diseases are independent of HDM or profilin sensitization. Our findings emphasize an important role of pollen sensitization in the manifestation of allergic disease in this region, which also suggests a need for primary prevention of sensitization against oil palm in this tropical environment of Southeast Asia.

## Abbreviations

A&E: accident and emergency department of a hospital; AD: atopic dermatitis; AR: allergic rhinitis; ARIA: Allergic Rhinitis Impact on Asthma; Can f: *Canis familiaris*; CI: confidence interval; Ela g Bd 31 K: 31-kDa palm pollen glycoprotein; Ela g: *Elaeis guineensis*; Fel d: *Felis domesticus*; GP: general practitioner; HDM: house dust mite; IPTG: isopropyl β-d-1-thiogalactopyranoside; ISAAC: International Study of Asthma and Allergies in Childhood; LB: Luria Bertani; NUS: National University of Singapore; OR: odds ratio; SMCSGES: Singapore and Malaysia cross-sectional genetic and epidemiological study; SPT: skin prick test; SSIgE: Serum specific IgE; SU: Sunway University; UTAR: University Tunku Abdul Rahman

## Funding sources

F.T.C. has received research support from the National University of Singapore, Singapore Ministry of Education Academic Research Fund, Singapore Immunology Network, National Medical Research Council (NMRC) (Singapore), Biomedical Research Council (BMRC) (Singapore), National Research Foundation (NRF) (Singapore), Singapore Food Agency (SFA), and the Agency for Science Technology and Research (A∗STAR) (Singapore); Grant No.: N-154-000-038-001, R-154-000-191-112, R-154-000-404-112, R-154-000-553-112, R-154-000-565-112, R-154-000-630-112, R-154-000-A08–592, R-154-000-A27–597, R-154-000-A91–592, R-154-000-A95–592, R-154-000-B99-114, BMRC/01/1/21/18/077, BMRC/04/1/21/19/315, BMRC/APG2013/108, SIgN-06-006, SIgN-08-020, NMRC/1150/2008, OFIRG20nov-0033, NRF-MP-2020-0004, SFS_RND_SUFP_001_04, W22W3D0006, H17/01/a0/008, and APG2013/108. K.R. has received funding from T20 Research Collaboration Grant Scheme from Sunway University with Grant No.: STR-RMF-T20-005-2019. Y.Y.S. has received research support from the NUS Resilience & Growth Postdoctoral Fellowships with Grant No.: R-141-000-036-281. All funding agencies had no role in the study design, data collection and analysis, decision to publish, or preparation of the manuscript.

## Data availability statement

All data used and included in this study are available from the corresponding author (Chew Fook Tim). Data is not publicly available due to ethical reasons. Further enquiries can be directed to the corresponding author.

## Author contributions

F.T.C. planned and supervised the study. Y.Y.S., G.A.V.N., J.A.L., S.A.M., Y.H.S., K.F.T., Y.R.W., S.M.R.S., and K.R. recruited participants for the study. Y.Y.S., G.A.V.N., and J.A.L. planned, conducted the experiments, and analyzed the data. Y.Y.S. wrote the manuscript. All authors reviewed and approved the manuscript.

## Statement of ethics

Participant recruitment study in Singapore was approved by the Institutional Review Board (IRB) in NUS. The study protocol was reviewed and approved by NUS-IRB, approval numbers: 07–023, 09–256, 10–445, 13–075, B-10-343, and H-18-036. Participant recruitment study in UTAR, Malaysia was approved by the UTAR Scientific and Ethical Review Committee (SERC). The study protocol was reviewed and approved by UTAR-SERC, approval number: U/SERC/03/2016. Participant recruitment study in SU, Malaysia was approved by the Research Ethics Committee at SU. The study protocol was reviewed and approved by the Research Ethics Committee at SU, approval number: SUREC 2019/029. All participants provided informed and written consent, with additional written informed consent obtained from the participant's parent, legal guardian, or next of kin for those below 21 years old. All recruitment procedures were carried out in concordance with the Helsinki Declaration.

## Authors’ consent for publication

All authors have read and consented to the publication of this manuscript.

## Declaration of competing interest

F.T.C. has received consulting fees from Sime Darby Technology Centre; First Resources Ltd; Genting Plantation, and Olam International, outside the submitted work. All funding agencies had no role in the study design, data collection, analysis, decision to publish, or preparation of the manuscript. The other authors declare no competing interests.

## References

[1] Venkatesan P. (2023). 2023 GINA report for asthma. Lancet Respir Med.

[2] Sacotte R., Silverberg J.I. (2018). Epidemiology of adult atopic dermatitis. Clin Dermatol.

[3] Savouré M., Bousquet J., Jaakkola J.J.K., Jaakkola M.S., Jacquemin B., Nadif R. (2022). Worldwide prevalence of rhinitis in adults: a review of definitions and temporal evolution. Clin Transl Allergy.

[4] Sio Y.Y., Pang S.L., Say Y.H. (2021). Sensitization to airborne fungal allergens associates with asthma and allergic rhinitis presentation and severity in the Singaporean/Malaysian population. Mycopathologia.

[5] Andiappan A.K., Puan K.J., Lee B. (2014). Allergic airway diseases in a tropical urban environment are driven by dominant mono-specific sensitization against house dust mites. Allergy.

[6] Chew F.T., Lim S.H., Shang H.S. (2000). Evaluation of the allergenicity of tropical pollen and airborne spores in Singapore. Allergy.

[7] Zhang L., Chew F.T., Soh S.Y. (1997). Prevalence and distribution of indoor allergens in Singapore. Clin Exp Allergy.

[8] Ong T.C., Lim S.H., Chen X. (2012). Fern spore and pollen airspora profile of Singapore. Aerobiologia.

[9] Sabit M., Wong C., Andaya A., Ramos J.D. (2020). Pollen allergen skin test and specific IgE reactivity among Filipinos: a community-based study. Allergy Asthma Clin Immunol.

[10] Alebiosu O.S., Adekanmbi O.H. (2022). Aerofloral studies and allergenicity of dominant pollen types in Taraba and Bauchi States of Northeastern Nigeria. Sci Total Environ.

[11] Xu Y., Yu L., Li W., Ciais P., Cheng Y., Gong P. (2020). Annual oil palm plantation maps in Malaysia and Indonesia from 2001 to 2016. Earth Syst Sci Data.

[12] Baxi S.N., Phipatanakul W. (2010). The role of allergen exposure and avoidance in asthma. Adolesc Med State Art Rev.

[13] Baratawidjaja I.R., Baratawidjaja P.P., Darwis A. (1999). Prevalence of allergic sensitization to regional inhalants among allergic patients in Jakarta, Indonesia. Asian Pac J Allergy Immunol.

[14] Kimura Y., Maeda M., Kimupa M. (2002). Purification and characterization of 31-kDa palm pollen glycoprotein (Ela g Bd 31 K), which is recognized by IgE from palm pollinosis patients. Biosci Biotechnol Biochem.

[15] Wang D., Chew F., Liew C., Tan H., Lee B. (2000). 981 Cloning and expression of an oil palm (Elaeis guineensis Jacq.) pollen allergen with homology to pectin esterases. J Allergy Clin Immunol.

[16] Hon, S., Lee, B., Tan, H. & Chew, F. in ALLERGY. 284-284 (WILEY-BLACKWELL 111 RIVER ST, HOBOKEN 07030-5774, NJ USA).

[17] Daengsuwan T., Lee B.W., Visitsuntorn N. (2003). Allergen sensitization to aeroallergens including Blomia tropicalis among adult and childhood asthmatics in Thailand. Asian Pac J Allergy Immunol.

[18] Leung R., Ho P., Lam C.W., Lai C.K. (1997). Sensitization to inhaled allergens as a risk factor for asthma and allergic diseases in Chinese population. J Allergy Clin Immunol.

[19] Wong Q.Y.A., Lim J.J., Ng J.Y. (2022). Allergic rhinitis in Chinese young adults from the Singapore/Malaysia cross-sectional genetics epidemiology study (SMCGES) cohort: Prevalence, patterns, and epidemiology of allergic rhinitis. World Allergy Organ J.

[20] Lim J.J., Lim Y.Y.E., Ng J.Y. (2022). An update on the prevalence, chronicity, and severity of atopic dermatitis and the associated epidemiological risk factors in the Singapore/Malaysia Chinese young adult population: A detailed description of the Singapore/Malaysia Cross-Sectional Genetics Epidemiology Study (SMCGES) cohort. World Allergy Organ J.

[21] Andiappan A.K., Sio Y.Y., Lee B. (2016). Functional variants of 17q12-21 are associated with allergic asthma but not allergic rhinitis. J Allergy Clin Immunol.

[22] Wong Q.Y.A., Lim J.J., Ng J.Y. (2023). An updated prevalence of asthma, its phenotypes, and the identification of the potential asthma risk factors among young Chinese adults recruited in Singapore. World Allergy Organ J.

[23] Lim S.H., Chew F.T., Mohd Dali S.D.B., Tan H.T.W., Lee B.W., Tan T.K. (1998). Outdoor airborne fungal spores in Singapore. Grana.

[24] Gao Y.F., Wang D.Y., Ong T.C., Tay S.L., Yap K.H., Chew F.T. (2007). Identification and characterization of a novel allergen from Blomia tropicalis: blo t 21. J Allergy Clin Immunol.

[25] Kidon M.I., Chiang W.C., Liew W.K. (2011). Mite component-specific IgE repertoire and phenotypes of allergic disease in childhood: the tropical perspective. Pediatr Allergy Immunol.

[26] Chan S.L., Ong T.C., Gao Y.F. (2008). Nuclear magnetic resonance structure and IgE epitopes of Blo t 5, a major dust mite allergen. J Immunol.

[27] Batard T., Hrabina A., Bi X.Z. (2006). Production and proteomic characterization of pharmaceutical-grade Dermatophagoides pteronyssinus and Dermatophagoides farinae extracts for allergy vaccines. Int Arch Allergy Immunol.

[28] Bousquet J., Khaltaev N., Cruz A.A. (2008). Allergic rhinitis and its Impact on asthma (ARIA) 2008 update (in collaboration with the world health organization, GA(2)len and AllerGen). Allergy.

[29] Asher M.I., Keil U., Anderson H.R. (1995). International study of asthma and Allergies in childhood (ISAAC): rationale and methods. Eur Respir J.

[30] Andiappan A.K., Anantharaman R., Nilkanth P.P., Wang de Y., Chew F.T. (2010). Evaluating the transferability of Hapmap SNPs to a Singapore Chinese population. BMC Genet.

[31] Asam C., Hofer H., Wolf M., Aglas L., Wallner M. (2015). Tree pollen allergens-an update from a molecular perspective. Allergy.

[32] Farahmand Fard M.A., Khanjani N., Arabi Mianroodi A., Ashrafi Asgarabad A. (2017). Asthma and allergic rhinitis correlation in palm tree workers of jahrom city in 2016. Iran J Otorhinolaryngol.

[33] Wang D.Y., Goh D.Y., Ho A.K., Chew F.T., Yeoh K.H., Lee B.W. (2003). The upper and lower airway responses to nasal challenge with house-dust mite Blomia tropicalis. Allergy.

[34] Asturias J.A., Ibarrola I., Fernández J., Arilla M.C., González-Rioja R., Martínez A. (2005). Pho d 2, a major allergen from date palm pollen, is a profilin: cloning, sequencing, and immunoglobulin E cross-reactivity with other profilins. Clin Exp Allergy.

[35] Asero R., Tripodi S., Dondi A. (2015). Prevalence and clinical relevance of IgE sensitization to profilin in childhood: a multicenter study. Int Arch Allergy Immunol.

[36] Scadding G.W., Calderon M.A., Shamji M.H. (2017). Effect of 2 Years of treatment with sublingual grass pollen immunotherapy on nasal response to allergen challenge at 3 Years among patients with moderate to severe seasonal allergic rhinitis: the GRASS randomized clinical trial. JAMA.

[37] Cipriani F., Calamelli E., Ricci G. (2017). Allergen avoidance in allergic asthma. Front Pediatr.

