# Supplementary material for: Sensitization to oil palm pollen associates with risks and severity of allergic diseases
Source: World Allergy Organ J. 2024 Jan 9;17(1):100853. doi: 10.1016/j.waojou.2023.100853 (PMC10792632; doi:10.1016/j.waojou.2023.100853)
Supplement: Multimedia component 1 [file mmc1.docx]

**
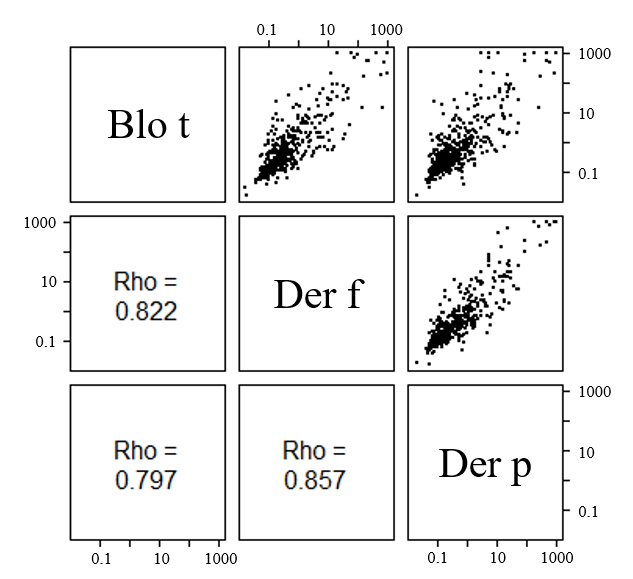
**

**Figure S1**: Pairwise correlation plot of serum specific IgE (SSIgE) titers against 3 common house dust mite allergens. **Upper panel**: pairwise correlation plots of SSIgE titers (IU/ml) against *Blomia tropicalis* (Blo t), *Dermatophagoides farinae* (Der f), and *Dermatophagoides pteronyssinus* (Der p). **Lower panel**: Spearman correlation coefficients (Rho) were shown. Results were assessed using *n*=564 serum samples from the SMCSGES cohort.

**
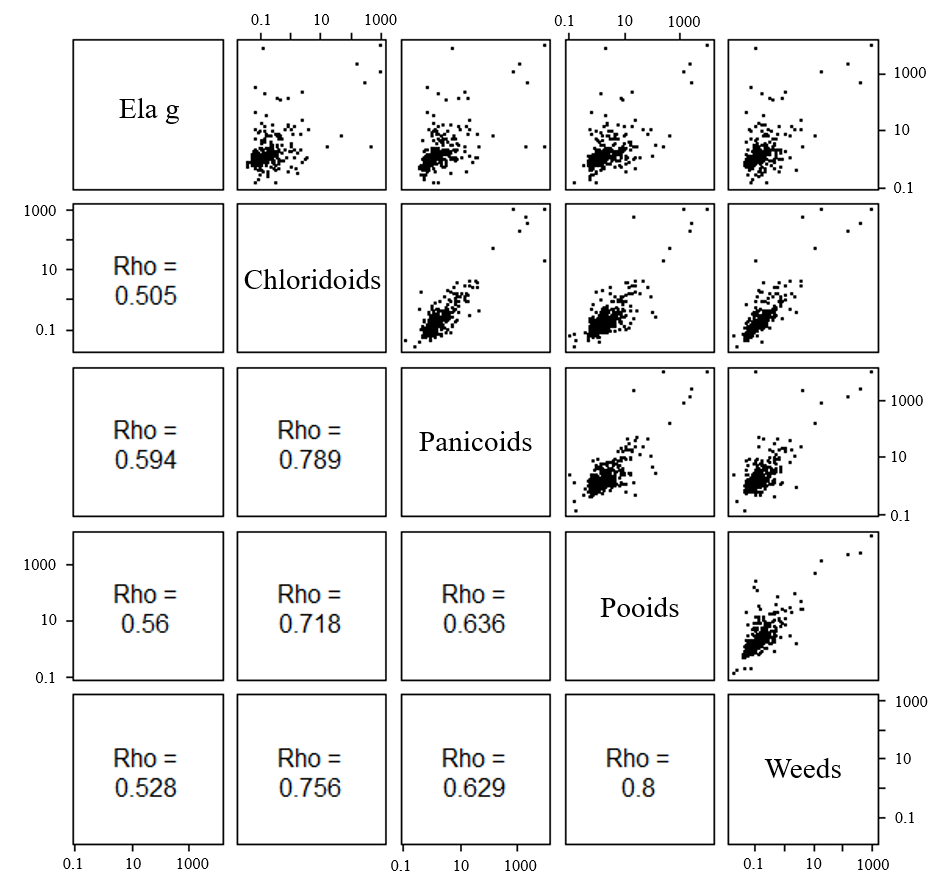
**

**Figure S2**: Pairwise correlation plots of serum specific IgE (SSIgE) titers against 5 common pollen allergens. **Upper panel**: pairwise correlation plots of SSIgE titers (IU/ml) against oil palm (*Elaeis guineensis*, Ela g), chloridoids (*Cynodon dactylon*), panicoids (*Sorghum halepense*), pooids (*Phleum pratense*, *Festuca pratensis*, and *Lolium perenne*), and weeds (*Brassica* spp., *Ambrosia artemisifolia*, and *Helianthus annus*) pollen allergens. **Lower panel**: Spearman correlation coefficients (Rho) were shown. Results were assessed using *n*=564 serum samples from the SMCSGES cohort.

**
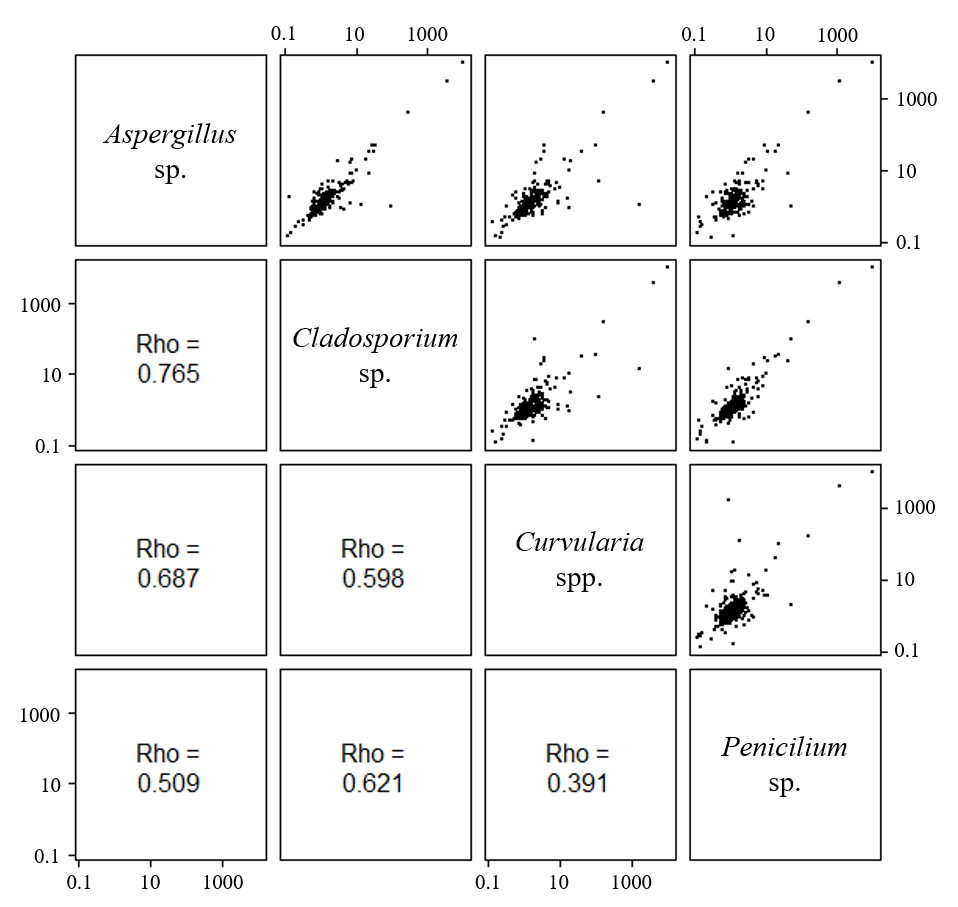
**

**Figure S3**: Pairwise correlation plots of serum specific IgE (SSIgE) titers against 4 common fungal allergens. **Upper panel**: pairwise correlation plots of SSIgE titers (IU/ml) against *Aspergillus* sp., *Cladosporium* sp., *Curvularia* sp., and *Penicilium* sp. allergens. **Lower panel**: Spearman correlation coefficients (Rho) were shown. Results were assessed using *n*=564 serum samples from the SMCSGES cohort.

**
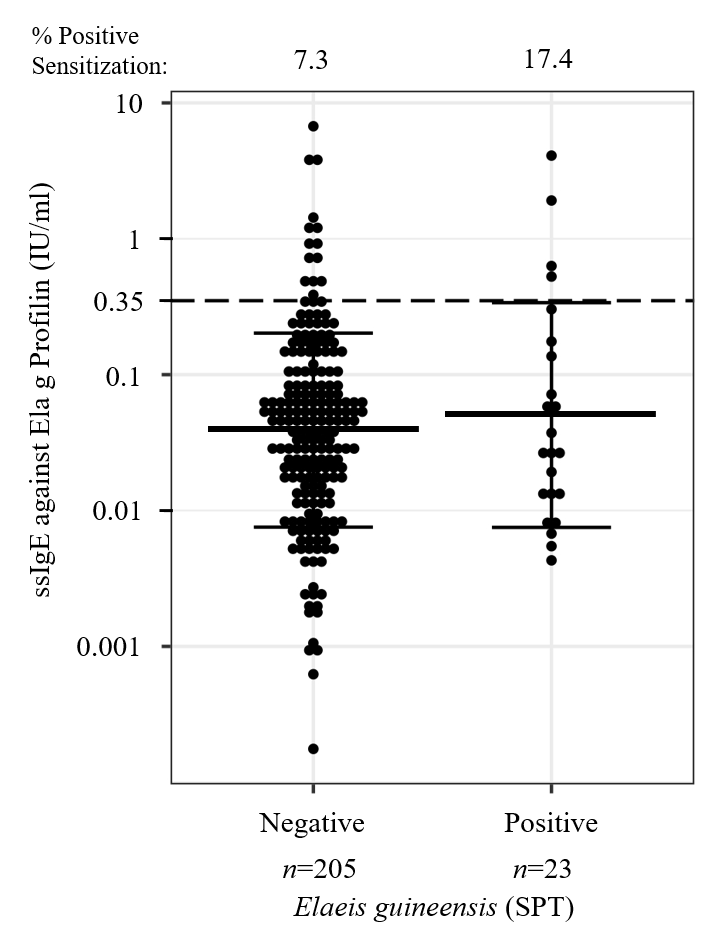
**

**Figure S4**: Frequency of sensitization against Ela g profilin in the Singapore/Malaysia population. Serum specific immunoglobulin-E (SSIgE) titers (as IU/ml) against Ela g profilin were assessed on *n*=228 Singapore/Malaysia young adults from the serological assessment cohort.

**Table S1.** Detailed demographics of the SMCSGES cohorts.

|  | Large-scale Epidemiological Assessment Cohort | Serological Assessment Cohort  (Assessment of SSIgE titers against inhalant allergens) |
| --- | --- | --- |
| **Total (*n*)** | 13,652 | 564 |
| **Gender**: Male (*n*, %) | 5659 (41.5) | 277 (49.1) |
| Female (*n*, %) | 7893 (57.8) | 270 (47.9) |
| **Age** (Year, Mean ± 1SD) | 21.96 ± 5.004 | 21.16 ± 3.49 |
| **Ethnicity:** Chinese (*n*, %) | 13,652 (100.0) | 516 (91.5) |
|  |  |  |
| **Single allergic disease (allergic rhinitis/atopic dermatitis/asthma)** | 4976 (36.4) | - |
| **More than one allergic disease (disease multimorbidity)** | 2470 (18.1) | - |
|  |  |  |
| **Asthma** (*n*, %) | 2405 (17.6) | - |
| **Allergic Rhinitis** (*n*, %) | 5453 (39.9) | - |
| **Atopic Dermatitis** (*n*, %) | 2447 (17.9) | - |
|  |  |  |
| **Sensitization (SPT positive)** |  |  |
| *Blomia tropicalis* | 7051 (43.2) | 277 (49.1) |
| *Dermatophagoides pteronyssinus* | 6580 (40.3) | 280 (49.7) |
| *Elaeis guineensis* | 647 (4) | 53 (9.4) |
| *Curvularia lunata* | 223 (1.4) | 11 (2.0) |
| At least one HDM* | 8089 (59.3) | 334 (59.2) |

HDM: house dust mite; SD: standard deviation; SPT: skin prick test, SSIgE: serum specific IgE. *refers to sensitization to at least one type of HDM allergens including *Blomia tropicalis* and *Dermatophagoides pteronyssinus*. Both cohorts are non-overlapping and independent of each other.

**Table S2.** Associations of *Elaeis guineensis* (oil palm) pollen sensitization with the severity and frequency of symptoms of atopic dermatitis (AD) in the Singapore/Malaysia Chinese population.

|  | Total  (*n* = 13,652) | |  |  |
| --- | --- | --- | --- | --- |
|  | *Elaeis guineensis*  Positive SPT | *Elaeis guineensis*  Negative SPT | *P*-value* | OR (95% CI) |
|  |  |  |  |  |
| **Severity of AD**† |  |  |  |  |
| Mild, *n* (%) | 80 (55.2) | 1019 (59.1) |  |  |
| Moderate, *n* (%) | 45 (31) | 513 (29.8) | 0.527 | 1.13 (0.77-1.65) |
| Severe, *n* (%) | 20 (13.8) | 192 (11.1) | 0.240 | 1.36 (0.79-2.24) |
| **Duration of AD symptoms**‡ |  |  |  |  |
| Persistent, *n* (%) | 94 (66.2) | 1075 (62.5) |  |  |
| Intermittent, *n* (%) | 48 (33.8) | 644 (37.5) | 0.425 | 0.86 (0.60-1.23) |

AD: atopic dermatitis; CI: confidence interval; OR: odds ratio; SPT: skin prick test. All data were evaluated based on skin prick test results of the *Elaeis guineensis* (oil palm) pollen allergen in a cross-sectional cohort of Singapore/Malaysia Chinese individuals (*n* = 13,652). *P*-value, odds ratio, and 95% CI were calculated using a logistic regression analysis with adjustment for age and gender.

*Logistic *p* < 0.05 is considered as significant.

†Severity of AD: defined as the frequency of test subjects kept awake at night due to the AD-related itchy rash (mild: never, moderate: less than one night per week, severe: at least one night per week).

‡Duration of AD symptoms: persistent symptoms refer to the AD-related itchy rash that did not clear completely in the past 12 months; intermittent symptoms refer to the same symptoms that cleared completely in the past 12 months.

**Table S3.** Frequency of serum specific IgE (ssIgE) sensitization against Ela g profilin among Singapore/Malaysia young adults with or without *Elaeis guineensis* (oil palm) pollen sensitization.

|  | Total | |  |  |
| --- | --- | --- | --- | --- |
|  | *Elaeis guineensis*  Positive SPT | *Elaeis guineensis*  Negative SPT | *P*-value* | OR (95% CI) |
|  |  |  |  |  |
| With detectable SSIgE to Ela g profilin (> 0.35 IU/ml), *n* (%) | 4 (17.4) | 15 (7.3) | 0.076 | 3.04 (0.79-9.78) |
| No detectable SSIgE to Ela g profilin (< 0.35 IU/ml), *n* (%) | 19 (82.6) | 190 (92.7) | Reference |  |

CI: confidence interval; OR: odds ratio; SPT: skin prick test; SSIgE: serum specific IgE. Data were evaluated based on serological assessment on a cohort of *n*=228 Singapore/Malaysia young adults from the serological assessment cohort. *P*-value, odds ratio, and 95% CI were calculated using a logistic regression analysis with adjustment for age and gender.

*Logistic *p* < 0.05 is considered as significant.
